# Software-Based Transformation of White Light Endoscopy Images to Hyperspectral Images for Improved Gastrointestinal Disease Detection

**DOI:** 10.3390/diagnostics15131664

**Published:** 2025-06-30

**Authors:** Chien-Wei Huang, Chang-Chao Su, Chu-Kuang Chou, Arvind Mukundan, Riya Karmakar, Tsung-Hsien Chen, Pranav Shukla, Devansh Gupta, Hsiang-Chen Wang

**Affiliations:** 1 Department of Gastroenterology, Kaohsiung Armed Forces General Hospital, 2, Zhongzheng 1st Rd., Lingya District, Kaohsiung 80284, Taiwan; forevershiningfy@yahoo.com.tw; 2 Department of Nursing, Tajen University, 20, Weixin Rd., Yanpu Township, Pingtung County 90741, Taiwan; 3 Division of Gastroenterology and Hepatology, Department of Internal Medicine, Ditmanson Medical Foundation Chia-Yi Christian Hospital, Chiayi 60002, Taiwan; 06155@cych.org.tw (C.-C.S.); vacinu@gmail.com (C.-K.C.); 4 Obesity Center, Ditmanson Medical Foundation Chia-Yi Christian Hospital, Chiayi 60002, Taiwan; 5 Department of Mechanical Engineering, National Chung Cheng University, 168, University Rd., Min Hsiung, Chiayi 62102, Taiwan; d09420003@ccu.edu.tw (A.M.); karmakarriya345@gmail.com (R.K.); 6 Department of Internal Medicine, Ditmanson Medical Foundation Chia-Yi Christian Hospital, Chiayi 60002, Taiwan; cych13794@gmail.com; 7 Department of Computer Science, Sanjivani College of Engineering, Station Rd., Singapur, Kopargaon 423603, Maharashtra, India; pranavshukla5467@gmail.com; 8 Computer Science and Engineering Department, Thapar Institute of Engineering & Technology, Patiala 147001, Punjab, India; dgupta1be21@thapar.edu; 9 Department of Medical Research, Dalin Tzu Chi Hospital, Buddhist Tzu Chi Medical Foundation, No. 2, Minsheng Road, Dalin, Chiayi 62247, Taiwan; 10 Hitspectra Intelligent Technology Co., Ltd., Kaohsiung 80661, Taiwan

**Keywords:** spectrum aided visual enhancer, wireless capsule endoscopy, gastrointestinal diseases, hyperspectral imaging, white light imaging, narrow-band imaging, polyps, oesophagitis, ulcerative colitis

## Abstract

**Background/Objectives:** Gastrointestinal diseases (GID), such as oesophagitis, polyps, and ulcerative colitis, contribute significantly to global morbidity and mortality. Conventional diagnostic methods employing white light imaging (WLI) in wireless capsule endoscopy (WCE) provide limited spectrum information, therefore influencing classification performance. **Methods:** A new technique called Spectrum Aided Vision Enhancer (SAVE), which converts traditional WLI images into hyperspectral imaging (HSI)-like representations, hence improving diagnostic accuracy. HSI involves the acquisition of image data across numerous wavelengths of light, extending beyond the visible spectrum, to deliver comprehensive information regarding the material composition and attributes of the imaged objects. This technique facilitates improved tissue characterisation, rendering it especially effective for identifying abnormalities in medical imaging. Using a carefully selected dataset consisting of 6000 annotated photos taken from the KVASIR and ETIS-Larib Polyp Database, this work classifies normal, ulcers, polyps, and oesophagitis. The performance of both the original WLI and SAVE transformed images was assessed using advanced deep learning architectures. The principal outcome was the overall classification accuracy for normal, ulcer, polyp, and oesophagitis categories, contrasting SAVE-enhanced images with standard WLI across five deep learning models. **Results:** The principal outcome of this study was the enhancement of diagnostic accuracy for gastrointestinal disease classification, assessed through classification accuracy, precision, recall, and F1 score. The findings illustrate the efficacy of the SAVE method in improving diagnostic performance without requiring specialised equipment. With the best accuracy of 98% attained using EfficientNetB7, compared to 97% with WLI, experimental data show that SAVE greatly increases classification metrics across all models. With relative improvement from 85% (WLI) to 92% (SAVE), VGG16 showed the highest accuracy. **Conclusions:** These results confirm that the SAVE algorithm significantly improves the early identification and classification of GID, therefore providing a potential development towards more accurate, non-invasive GID diagnostics with WCE.

## 1. Introduction

GID covers a wide spectrum of diseases affecting the oesophagus, stomach, small intestine, colon, and related organs, therefore influencing the natural structure and operation of the digestive tract [1,2,3]. From benign growths and inflammatory diseases to cancers, these disorders can range in degree of severity and clinical consequences. Among the most often seen GIDs are ulcers, polyps, and oesophagitis; normal mucosal variances form the basis for diagnostic comparisons [4,5,6,7,8]. Ulcers are usually linked to Helicobacter pylori infection or too much release of gastric acid; they are localised erosions of the mucosal surface [9,10]. By compromising the integrity of the gastrointestinal lining, these lesions can cause problems including stricture development, bleeding, and perforation [11]. Conversely, polyps are mucosal outgrowths that, especially in cases of adenomatous polyps, may stay benign or develop into malignant tumours; hence, early identification is very important to prevent colorectal cancer [12,13,14,15]. With symptoms including dysphagia and chest discomfort, oesophagitis, an inflammatory disorder of the oesophageal lining, can be brought on by anything from acid reflux, infection, or medication-induced harm [16]. Timely and suitable clinical action depends on accurate distinction between these pathogenic states and normal mucosa. Early diagnosis of GID is important since many of them are asymptomatic in their first stages and only show clinically once significant tissue damage has occurred [17,18,19]. Early-stage detection greatly increases prognosis, lowers treatment complexity, and lowers healthcare costs in disorders including colon cancer and inflammatory bowel disease. Nevertheless, the generic character of symptoms, overlapping morphological traits among several illnesses, and the limits of traditional imaging techniques can hinder the diagnosis process. Visual inspection by itself usually does not find minor mucosal changes or subepithelial changes before the expression of overt illness. Moreover, the anatomical complexity and great length of the GI tract provide major difficulties for thorough analysis by conventional methods. The medical community is thus always looking for cutting-edge imaging technology that can offer more exact classification of GI anomalies, better vision, and higher spectral resolution.

In this field, wireless capsule endoscopy (WCE) has become a revolutionising solution [20,21]. This minimally invasive diagnostic equipment consists of swallowing a tiny capsule including a wireless transmitter, light source, battery, and tiny camera and camera lens. Thousands of photos are captured by the capsule as it passes the digestive tract using natural peristalsis and sent to an external receiver carried by the patient. Clinicians then go over the gathered information, looking for anomalies. WCE’s main benefits are its non-invasiveness, patient comfort, and capacity to reach small intestine areas outside the reach of conventional endoscopes. It is a sensible and patient-friendly alternative since it removes the need for anaesthesia and makes outpatient screening possible. WCE is hampered, nonetheless, by many technical limitations, notwithstanding its simplicity. The capsule’s limited battery life limits the length of imaging, which sometimes results in partial inspection of the whole gastrointestinal system [22]. Although good for general viewing, the image resolution is less than that of high-definition conventional endoscopy. Furthermore, the technique produces a great amount of image data, which presents major difficulties for hand interpretation and calls for the employment of automated analysis tools. WCE, most importantly, depends on white light imaging (WLI), which lacks the spectrum richness required for comprehensive tissue characterisation and takes images in the usual red, green, and blue (RGB) channels [23,24]. Both conventional endoscopy and capsule endoscopy technologies still most often use WLI. It works by shining broad-spectrum white light on the mucosa and gathering reflected light using RGB sensors. Although this method offers a natural-colour view of the mucosal surface, it is not very good at distinguishing between tissue types, particularly in the early phases of illness. WLI’s broad spectra average out fine spectral variations that might be important for differentiating between healthy, inflammatory, and malignant tissues. Diagnostic sensitivity and specificity are thereby impaired, especially in the evaluation of flat or mild lesions. The approach’s dependence on visible colour patterns adds even more variation depending on lighting conditions, mucosal reflectivity, and physician experience. WLI is still rather helpful for general endoscopic study, but its use in complex tissue classification and early-stage pathology identification is under doubt more and more. Narrow band imaging (NBI) was designed to improve mucosal and vascular contrast without the necessity of dyes or chemical agents to overcome the limits of WLI [25,26]. Using optical filters, NBI limits illumination to two wavelengths, 415 nm in the blue spectrum and 540 nm in the green spectrum, that match the haemoglobin absorption maxima. NBI improves the visibility of superficial blood vessels and mucosal patterns by stressing these wavelengths, therefore helping to identify anomalies that could be missed under conventional white light [27]. This improved contrast helps to identify neoplastic alterations like aberrant angiogenesis linked with tumours. NBI has certain limitations [28]. Particularly on WCE systems, its implementation calls for specialised optical hardware not found in all endoscopic systems. Moreover, it still has a limited spectral range, which makes it insufficient for biochemical tissue differentiation, even if it gives better visualisation of vascular structures. NBI thus acts as a complement to WLI rather than a substitute; its diagnostic power is still limited by the physical constraints of its spectrum selection. By recording thorough spectrum information across hundreds of small, contiguous wavelength bands, hyperspectral imaging (HSI) offers a more complete method of biological imaging [29]. Every pixel in an HSI image has a complete reflectance spectrum, which helps to extract morphological and biochemical data beyond what RGB or NBI systems can provide. This lets one identify tissue kinds, diseases, and physiological changes depending on their distinct spectral signatures. From agricultural to environmental monitoring to, more recently, medical diagnostics, HSI has shown considerable promise in several disciplines. HSI is a useful technique for early illness diagnosis in GI imaging since it can expose minute variations in tissue oxygenation, water content, and cellular composition. But frequently large, costly, and complicated, HSI systems need exact calibration, great processing capability, and plenty of storage space. Particularly in WCE, poor capture rates and the requirement for specialised equipment impede real-time deployment in clinical situations. These restrictions have thus far hindered the general acceptance of HSI in standard endoscopic procedures.

Understanding the complementary strengths of NBI and HSI, a new computational method called the Spectrum-Aided Vision Enhancer (SAVE) synthesises their benefits into a single, software-driven solution [30]. Designed to translate traditional WLI obtained from regular endoscopic or capsule-based systems into improved spectral representations that mimic both HSI and NBI, the SAVE method. This is accomplished by means of a multi-stage approach comprising rigorous spectrum calibration with a Macbeth Colour Checker, principal component analysis (PCA) for spectral reconstruction, and limited band selection to mimic NBI wavelengths [31]. SAVE generates synthetic HSI data from conventional RGB inputs using mathematical modelling and machine learning (ML) methods; next, band filtering replicates NBI-enhanced images as shown in Table 1. To guarantee high-fidelity spectrum reconstruction, the method additionally combines colour correction, gamma linearisation, and light-source spectral matching. SAVE is compatible with current imaging platforms, including wireless capsule endoscopes, by allowing this capability through software, therefore overcoming the need for additional hardware components. SAVE’s driving force comes from the vital necessity to close the gap between hardware constraints and diagnostic potential in present GI imaging systems. NBI improves structural contrast while HSI provides unmatched spectral detail; both are hampered by equipment limitations that restrict their accessibility and scalability. SAVE solves this by converting easily available WLI data into a format that preserves the diagnostic ability of more sophisticated imaging modalities. This method not only provides democratisation of access to improved diagnostics but also creates opportunities for integration with artificial intelligence systems capable of automatic disease classification. By accomplishing this, SAVE prepares the path for a new generation of clinically significant, resource-efficient, image-based diagnostic instruments. SAVE has the potential to greatly progress in the field of gastrointestinal diagnostics and help to improve patient outcomes worldwide by means of enhanced picture contrast, improved classification accuracy, and early illness detection.

## 2. Materials and Methods

This is a retrospective, observational study utilising publicly available datasets to evaluate the effectiveness of the SAVE method in improving diagnostic accuracy for gastrointestinal disease classification. This study aims to compare the efficacy of our deep-learning models in differentiating normal tissue, ulcers, polyps, and oesophagitis using both the standard WLI and SAVE-enhanced images. The principal metric was overall classification precision, represented as the frequency of accurately predicted labels. To assess the models’ reliability and comprehensiveness, we analysed recall (the number of correctly identified true cases). Ultimately, we computed the F1 score to equilibrate precision and recall, derived the mean average precision to encapsulate performance across various decision thresholds, and examined confusion matrices to identify any systematic misclassification patterns.

### 2.1. Dataset

In this study, a curated dataset comprising GI tract images was utilised to train and evaluate the performance of the SAVE algorithm. Two publicly available, well-established datasets, KVASIR and ETIS-Larib Polyp Database, were combined to create a new dataset [36]. Designed for GID categorisation and detection, the extensive multi-class picture repository known as the KVASIR dataset was created by Vestre Viken Health Trust and Simula Research Laboratory. It comprises endoscopic pictures taken during standard tests under the direction of professional gastroenterologists [37]. These pictures show different pathological and normal findings, including ulcers, polyps, oesophagitis, and normal mucosa. The ETIS-Larib Polyp Database offers more annotated pictures, especially targeted at polyp recognition, therefore augmenting the KVASIR dataset. The combined dataset was randomly shuffled and stratified into three subsets: training (53.3%), validation (33.3%), and testing (13.4%), therefore guaranteeing the robustness of the classification models and avoiding data leakage. There were 6000 images overall, split as follows: 800 for testing, 2000 for validation, and 3200 for training [38]. Every one of the four target classes, normal, ulcer, polyp, and oesophagitis, was equally distributed among the three splits, therefore guaranteeing class balance and removing evaluation bias. Table 2 lists image distribution per class and data split. In this study, the dataset was partitioned into three subsets: training (70%), validation (20%), and testing (10%). The training set supplied adequate data for model learning, whereas the validation set was utilised for hyperparameter optimisation and overfitting surveillance. The test set facilitated an impartial assessment of model efficacy. Cross-validation, while a valuable technique, was not utilised due to computational constraints that necessitate multiple training iterations, thereby substantially augmenting computational time and resources, particularly with deep learning models. The dataset was sufficiently large to yield reliable performance estimates with the train-validation-test split, rendering cross-validation superfluous. This study aimed to evaluate the model’s generalisation capability, and the train-validation-test split effectively fulfilled this objective.

**Figure 1:**
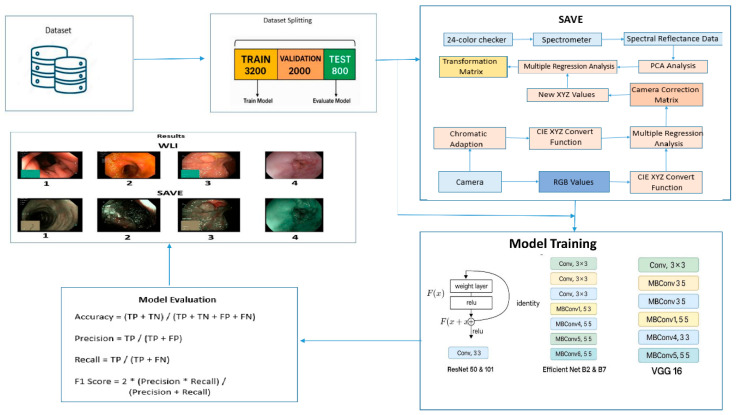
Workflow of the study.

Consistent with the input criteria of the deep learning architectures applied in this work, all photos were resized to a fixed dimension of 224 × 224 pixels. Using TensorFlow’s Keras’ Image Data Generator class, image scaling was done that not only allowed effective image loading but also reduced computational cost during training. Crucially, considerable data augmentation was used on the training set to improve model generalisation and stop overfitting before training the deep learning models. Among the augmenting methods were random rotation, horizontal and vertical flipping, zooming, and shearing. While varying the feature space, these changes maintained the anatomical integrity of the GI components. Especially, no augmentation was done to the test and validation sets to guarantee that model performance was assessed on unprocessed, fresh data (see Supplement Materials Table S1 for the hyperparameters of the models used). All photos were pre-processed using standard normalising methods and kept in the RGB colour space. Pixel values were scaled to a [0, 1] range, and during model training, additional changes like noise regularisation and gamma correction were applied. The suggested system depends critically on the whole process of preparing a dataset. From dataset preparation and image enhancement using SAVE to classification using advanced convolutional neural networks (CNNs) and last evaluation using performance metrics including accuracy, precision, recall, and F1 score, it feeds directly into the workflow shown in Figure 1 (see Supplement Materials Section S1 for more details about the dataset).

### 2.2. Spectrum Aided Vision Enhancer

RGB values, which result from varying intensities of red, green, and blue components, allow every colour that the human eye can see to be portrayed. But the HSI colour model adds the intensity of light absorption and reflection, so providing a more complex knowledge of colour properties and extending this representation. By means of the development of a reflectance chart, the SAVE program transforms digital images taken by RGB cameras into the HSI domain. Using a Macbeth Colour Checker (X-Rite Classic), a set of 24 colour swatches comprising primary colours (red, green, blue), secondary colours (cyan, magenta, yellow), and six greyscale tones, this conversion is calibrated.

The 24-colour patch images are converted into the CIE 1931 XYZ colour space, which linearises and normalises the RGB inputs, thereby more nearly approximating human colour perception. Applied is a variable matrix correction approach, stated in Equation (1), since digital camera sensors are prone to capturing noise and may introduce systematic inaccuracies. After rectification, XYZ values are then obtained as shown in Equation (2). While reflectance data from a spectrometer experiences a similar change via Equations (3)–(6), this technique allows consistent mapping of sRGB data from camera pictures to the XYZ colour space.

(1) $$\left[C\right]=\left[{XYZ}_{Spectrum}\right]\times pinv(\left[V\right])$$

(2) $$\left[{XYZ}_{Correct}\right]=\left[C\right]\times [V]$$

(3) $$X=k\int_{400\;nm}^{700\;nm}S\left(\lambda \right)R\left(\lambda \right)\bar{x}\left(\lambda \right)d\lambda$$

(4) $$Y=k\int_{400\;nm}^{700\;nm}S\left(\lambda \right)R\left(\lambda \right)\bar{y}\left(\lambda \right)d\lambda$$

(5) $$Z=k\int_{400\;nm}^{700\;nm}S\left(\lambda \right)R\left(\lambda \right)\bar{z}\left(\lambda \right)d\lambda$$

(6) $$k=100/\int_{400\;nm}^{700\;nm}S\left(\lambda \right)\bar{y}\left(\lambda \right)d\lambda$$

During calibration, a preset dark current offset included in the imaging hardware is incorporated. Standardising the outputs of the V_colour_, V_non-linear_, and V_dark_ matrices yields the final transformation matrix, limited to a third-order polynomial that helps to prevent overfitting. To create baseline XYZ values, the Ocean Optics QE65000 (Ocean Optics, Orlando, FL, USA) spectrometer records the spectral reflectance of all 24 colour patches in concert with the X-Rite colour chart. By computing a customised transformation matrix (M) in Equation (7), a regression-based optimisation procedure then reduces conversion discrepancies and offsets sensor-specific properties. Later, a regression phase helps to further match the expected XYZ values with reference data, thereby improving calibration precision.

(7) $$\left[M\right]=\left[Score\right]\times pinv(\left[V_{Colour}\right])$$

From which a similarity index is produced to evaluate the fit between the spectrometer and algorithm estimated XYZ outputs, the reflectance spectrum (RSpectrum) is fundamental in this process. Applying PCA to RSpectrum reveals six main components that account for 99.64% of the total spectral variance. Under controlled lighting, the final RGB to XYZ translation is optimised using the conventional CIE 1931 metrics to solve spectral responses particular to cameras. High chromatic accuracy was obtained by a significant correlation between the transformation matrix and PCA components, producing an average colour deviation of 0.75 and a root mean square error (RMSE) of 0.056. Especially, the average chromatic aberration fell greatly from 10.76 to 0.63 post-calibration (see Supplement Materials Table S2 for the RMSEs of the XYZ values before and after calibration). While 23 of the 24 colour blocks had RMSE values below 0.1, with black recording the lowest at 0.015, red tones showed the highest inaccuracy in the 600–780 nm range (see Supplement Materials Figure S11 for the colour difference before and after camera calibration).

The SAVE approach uses wavelength-specific enhancement to expose damaged areas more clearly, so permitting earlier diagnosis given the limits of standard WLI in diagnosing GID (see Supplement Materials Figure S12 for the RMSEs between analogue and measured spectra of each colour block). HSI processing transforms RGB images into NBI-like images, tuned for Olympus endoscopic devices. Using CIEDE 2000 measurements, the SAVE-generated images are colour-matched with real NBI visuals, therefore producing a minimum colour difference of 2.79 (see Supplement Materials Figure S13 for the LAB values of the simulated and observed colours). Using the Cauchy-Lorentz distribution, further optimised by Fast Simulated Annealing (FSA), discrepancies resulting from changes in the reflection spectrum, lighting, and colour matching function were addressed in Equation (8). The residual colour difference was reduced to 5.36 after rectification, a level judged negligible to the human eye.

(8) $$f\left(x;x_{0},\gamma \right)=\frac{1}{\pi \gamma \left[1+{\left(\frac{x-x_{0}}{\gamma }\right)}^{2}\right]}=\frac{1}{\pi }\left[\frac{\gamma }{{\left(x-x_{0}\right)}^{2}+\gamma^{2}}\right]$$

Integration of wavelengths at 600 nm, 700 nm, and 780 nm also helped to account for artefacts and improved skin tissue representation, especially in the presence of brown pigmentation at 650 nm—a departure from usual haemoglobin absorption maxima at 415 and 540 nm. These enhancements made synthetic NBI images more visually akin to actual ones. Confirming the great authenticity and efficiency of the SAVE technique for advanced medical imaging uses, quantitative tests revealed an average picture entropy of 0.37%, a Structural Similarity Index (SSIM) of 94.27%, and a Peak Signal to Noise Ratio (PSNR) of 27.88 DB.

### 2.3. Model Architecture

Despite the recent prominence of deep learning architectures like Vision Transformers (ViTs), this study opted to utilise established models such as EfficientNet, ResNet, and VGG for various reasons. These models demonstrate robust performance across diverse image classification tasks, including medical imaging, and provide a dependable baseline for comparison. Their computational efficiency and stability render them appropriate for our study, guaranteeing a balance between performance and resource limitations. The originality of this study resides not in the selection of these models but in the implementation of the SAVE technique. SAVE transforms conventional WLI into HSI-like representations, markedly enhancing diagnostic precision without requiring specialised equipment. By incorporating this innovative image enhancement technique with prevalent deep learning models, we seek to present a practical and efficient solution for enhancing the early detection of gastrointestinal diseases.

#### 2.3.1. EfficientNetB2

EfficientNetB2, a member of the Efficient Net family, is used for GID classification [39]. Originally motivated by Mobile Net, the model design includes mobile inverted bottleneck convolutional blocks [40]. Different regularisation methods, including batch normalisation, Gaussian noise, dropout, and thick layers, were combined into the design to improve generalisation and reduce overfitting. Load and enhance the dataset using Keras’ ImageDataGenerator, then apply transformations like rotation, zoom, shear, and horizontal flips. Every image was cropped to 224 × 224 pixels. Input dimensions of 224 × 224 × 3 led the EfficientNetB2 model to be started with pre-trained ImageNet weights [41]. The initial classification layers were eliminated to allow for the integration of custom layers catering to the work. The pre-trained EfficientNetB2 base comprised the first layer; next, a Gaussian noise layer with a noise factor of 0.35 would help to improve generalisation. The spatial dimensions of the feature maps were lowered using a Global Average Pooling 2D layer; then, ReLU activation and a dense layer with 256 units followed. Standardising feature distributions by means of batch normalisation enhanced convergence. To prevent overfitting, a dropout layer was included; the last layer, for multi-class classification, was a dense layer of four units. The loss function for the model was categorical cross-entropy, and the Adam optimiser was compiled. Early halting was used with a 300-epoch patience to stop overtraining. Model Checkpoint helped to save the model with the lowest validation loss, hence improving model performance. Using training, validation, and test datasets, the trained model was tested for classification performance measured by metrics including precision, recall, and accuracy obtained from the confusion matrix and classification report.

#### 2.3.2. EfficientNetB7

EfficientNetB7 is the most powerful model in the Efficient Net family. It uses a compound scaling method [42]. Efficient Net scales three dimensions simultaneously for achieving the best performance. It is the most computational and the most expensive, but it has the highest accuracy. The EfficientNetB7 is built on the mobile inverted bottleneck convolution (MBConv) layer, which is more efficient than traditional convolutions and helps reduce computational cost without any compromise in accuracy. While training this model, augmentation was done only on the training dataset using techniques like rotation, shear, zoom, horizontal shift, and vertical shift for artificially increasing the dataset size and variability. But there was no augmentation performed on validation and training. The flow from the directory was used to load the dataset in batches. EfficientNetB7 is used for feature extraction, which first custom classifier layer present on top. Gaussian noise is added with a standard deviation of 0.35 to maintain better generalisation. The next layer was of dense layer with 256 units and ReLU activation [43]. It was used for learning the complex patterns of the dataset. For preventing overfitting, we have applied a kernel regularizer with L2regularizers with 0.01. Batch Normalisation was used for normalising the output of the previous layer and stabilising the learning process. Again, Custom Gaussian Noise was added to further regularise the model. A dropout layer of 0.5 was added to prevent overfitting the random output units to zero during training. The last layer was of dense layer consisting of 4 units for multi-class classification.

#### 2.3.3. ResNet 50

ResNet50 is part of Residual Networks, whose basic idea is a residual learning framework, and it makes the training of deeper networks using skip connections or shortcut connections that skip one or more layers [44]. It helps with the vanishing gradients problem that arises when we train very deep layers. It consists of 50 layers and is used to train very deep models using residual blocks, which increase training efficiency. In this work, we have used this model for the classification of GID. ImageDataGenerator was used for augmenting the training dataset and to generate batches of datasets. Train generator, validation generator, and test generator WERE used to load datasets from the directories. In this model, the target size of images was 224 × 224 pixels, and the batch size was 32. The Gaussian Noise layer was extended to add custom behaviour and noise during regulation to prevent overfitting. In this, we have trained ResNet50 on the ImageNet dataset. The architecture starts with building a Sequential width, creating an empty neural network. After that, we add the ResNet50 as the feature extractor. Gaussian noise is added with a standard deviation of 0.35. The next layer is GlobalAveragePooling2D for reducing the output from the base model to a single vector. We have used a dense layer to add a fully connected layer with 256 units with the ReLU activation. The next layer was added of the L2 regularisation to the dense layer for preventing overfitting. Then normalise output using Batch Normalisation. Dropout layers randomly drop 50% of the neurons to prevent overfitting. The last layer was a dense layer consisting of 4 units for multi-class classification.

#### 2.3.4. ResNet 101

ResNet101 uses residual learning to identify the shortcut for skipping one or more layers. It contains 101 layers of convolutional layers, batch normalisation, and ReLU activation. This model architecture is known for classification tasks and uses the combination of pre-trained machine learning models, custom layers, and different regularisation techniques. The backbone of this model is a pre-trained ResNet101 architecture that has been fine-tuned for extracting the features. It has been introducing a custom Gaussian noise layer at first, which has a standard deviation of 0.35. It helps with regularisation and control of overfitting. The next layer is a GlobalAveragePooling2D layer, which is used for reducing the spatial dimensions while retaining the essential information. Additionally, dense layers with 256 neurons and with ReLU activation are added with L2 regularisation of 0.01 for reducing overfitting. Batch normalisation was applied for stabilising and accelerate the training. Another Gaussian noise layer with a dropout layer of 0.5 ensures that the model is generalising well and avoiding overfitting. The output layer, or the last layer, has four units with a SoftMax activation function, making it suitable for multi-class classification. For training, we used the Adam optimiser with a learning rate of 0.0001. The model was trained for 300 epochs, and it had early stopping with a learning rate reduction technique used for preventing overfitting and ensuring the model is not stuck.

#### 2.3.5. VGG 16

VGG16 is a deep network that consists of 16 layers, out of which 13 are convolutional layers and 3 are fully connected layers [45]. It uses small 3 × 3 convolution filters that have a stride of 1 and padding for preserving the spatial dimensions and ensuring detailed feature extraction. In this, VGG16 was trained on the pre-trained ImageNet dataset. VGG16 has been used as the base model. This architecture has the custom Gaussian noise layer added after the base model for regularising and reducing overfitting; it has a standard deviation of 0.35 and is responsible for adding the noise to the inputs during training. The next layer is the global average pooling layer, which is used for reducing the spatial dimensions of feature maps, and the output has a fixed-length vector. Additionally, dense layers with 256 neurons and with ReLU activation are added with L2 regularisation of 0.01 for reducing overfitting. Batch normalisation was applied for stabilising and accelerate the training. Another Gaussian noise layer with a dropout layer of 0.5 ensures that the model is generalising well and avoiding overfitting. The output layer, or the last layer, has four units with a SoftMax activation function, making it suitable for multi-class classification. For training, we used Adam optimiser with a learning rate of 0.0001. The model was trained for 300 epochs, and it has early stopping with a learning rate reduction technique used for preventing overfitting and ensuring the model is not stuck.

### 2.4. Evaluation Metrics

The evaluation metrics included in this particular study are Precision (P), Recall (R), mean Average Precision (mAP), F1-score and confusion matrix. Some of the concepts associated with these metrics are True Positive (TP), False Positive (FP), and False Negative (FN). TP, just as the name suggests, are correct detections of the ground truth bounding box. While FP are incorrect detections of a nonexciting class or misplaced detections of an object, and FN are simply undetected ground-truth bounding boxes.

Precision is an evaluation metric that measures the number of instances that were correctly predicted. It is given by Equation (9), as follows:

(9) $$P=(\frac{TP}{TP+FP})\times 100$$

A high precision value indicates that the model has a low rate of false positives. Recall is known as a positive rate and is the probability of actual positive instances that the model identifies correctly, which is given by Equation (S2).

(10) $$R=(\frac{TP}{TP+FN})\times 100$$

F1-score is a measure of predictive performance. It is calculated based on the precision and recall of the model and is given by Equation (11).

(11) $$F1=\frac{2\times Precision\times Recall}{Precision+Recall}$$

The area under the precision-recall curve for a specific class in the precision vs. recall graph plotted for different threshold values is the average precision (AP). mAP is the mean of Aps and is given by Equation (12), as follows:

(12) $$AP=\int_{0}^{1}Precision(Recall)dRecall$$

## 3. Results

Using a publicly available dataset of 6000 GI endoscopic images, the efficacy of the suggested SAVE approach was assessed. To ensure the precision of the annotations, a selection of images from each category, including ulcers and oesophagitis, was evaluated by expert gastroenterologists. This validation process verified the accuracy of the labels for these images. It is crucial to acknowledge that not all images in the dataset received expert validation, which may be regarded as a limitation of the study. Notwithstanding this, we assert that the annotations in the public datasets are predominantly dependable for training objectives. Three subsets, 3200 images for training, 2000 for validation, and 800 for testing, were formed out of this dataset. Five separate machine learning classifiers were trained and evaluated using both SAVE-converted photos and conventional WLI. Standard evaluation measures, including precision, recall, and F1 score, were used in a thorough performance comparison. Overall, the experimental findings unequivocally showed that the SAVE technique regularly beat conventional WLI. Especially in complicated diagnostic situations, this performance improvement emphasises the possibilities of spectrally enhanced imaging in raising the classification accuracy of GID.

### 3.1. Efficient Net B2

The EfficientNetB2 model’s SAVE attained an accuracy of 97%, while WLI achieved 96%. An increase of 1% in accuracy was noted, as the F1 score improved across all classes, indicating more accurate detection compared to WLI. The precision results indicate that the three classes—normal, polyps, and oesophagitis—exhibit a higher true positive detection rate than WLI. In the ulcer class, we observed a 1% decrease in precision and a 5% increase in recall, indicating an increase in false positive predictions and a decrease in false negatives during the classification of this class in SAVE. Oesophagitis was accurately classified, achieving 100% accuracy across all parameters. The classification results are presented in Table 3 (see Supplement Materials, Figures S1 and S2, for the confusion matrix and classification report of WLI and SAVE EfficientNet B2).

### 3.2. Efficient Net B7

The EfficientNetB7 model has attained the highest accuracy in SAVE, which is 98%. This increase was noted due to the rise in F1 score across three classes: normal, ulcer, and oesophagitis, indicating that these classes were accurately identified. The same observation noted in the ulcer class of EfficientNetB2 was evident here, with the only difference being that accuracy was greater compared to EfficientNetB2. In polyps, it was noted that the contrary of what we observed in ulcers. There was a 1% reduction in false positives; however, false negatives increased by 2%, maintaining an accuracy of 96% in both precision and recall. The classification results are presented in Table 4 (see Supplement Materials, Figures S3 and S4, for the confusion matrix and classification report of WLI and SAVE EfficientNet B7).

### 3.3. ResNet 50

The ResNet50 model SAVE exhibits a 3% improvement relative to WLI. The F1 score of SAVE increased among the three classes: ulcer, polyps, and oesophagitis, while the normal class remained unchanged. The normal class exhibits a 1% reduction in precision rate relative to WLI, indicating that WLI has identified 1% more true positives. The ulcer exhibits a consistent pattern; however, there is a 21% increase in the recall rate compared to WLI, indicating that SAVE has reduced false negatives by 21%, resulting in 42 images being accurately classified in comparison to WLI. The polyps exhibit a consistent pattern; however, the recall decrease was more pronounced in this instance compared to the other model, which was 12%. The classification results are presented in Table 5 (see Supplement Materials, Figures S5 and S6 for the confusion matrix and classification report of WLI 7 SAVE ResNet 50).

### 3.4. Resnet 101

The ResNet101 model exhibited a 4% increase in performance in the SAVE compared to WLI. The accuracy improved due to the increase in the F1 score of the SAVE dataset across all classes compared to WLI. The normal class and oesophagitis class achieved a classification accuracy of 100%, indicating that all 200 images were accurately classified. In ulcer detection, precision, recall, and F1-score all achieved a rate of 93%, while the false negative classification decreased by 18%. It was observed that the false negative classification of polyps decreased by 12%, equating to 24 images. The classification report is presented in Table 6 (see Supplement Materials, Figures S7 and S8 for the confusion matrix and classification report of WLI 7 SAVE ResNet 101).

### 3.5. VGG16

The VGG16 model was trained on both the SAVE and WLI datasets. The most significant increase observed in this model was 7% in the SAVE. The F1 score is crucial, demonstrating enhanced accuracy across all classes in SAVE, which signifies an improvement in true classification. The recall rate for the ulcer class rose from 56% to 84%, indicating that SAVE has reduced false negatives by 28% compared to WLI, which corresponds to 56 images. The precision results indicate that SAVE predicted fewer false positives across all three classes: normal, polyps, and oesophagitis. The classification results are presented in Table 7 (see Supplement Materials, Figures S9 and S10, for the confusion matrix and classification report of WLI and SAVE of the VGG16 model).

## 4. Discussion

This study’s principal innovation is the creation of the SAVE, a groundbreaking pre-processing method that converts conventional WLI into hyperspectral-like formats. In contrast to current research that predominantly utilises pre-trained models or transformer-based techniques, the SAVE method augments the spectral information in WLI images, emulating NBI and amplifying the absorption characteristics of haemoglobin. This enhancement facilitates superior visualisation of gastrointestinal structures, resulting in more precise detection and classification of conditions such as ulcers, polyps, and oesophagitis. To our knowledge, no other studies have utilised a comparable HSI conversion algorithm to transform WLI images into an NBI-like structure for enhanced disease detection. The SAVE method offers a distinctive advancement in the field by augmenting the input data itself, rather than exclusively depending on deep learning models or image processing techniques. This pre-processing step can substantially enhance the efficacy of deep learning models in VCE, rendering it an invaluable asset for non-invasive gastrointestinal diagnostics. The SAVE method exhibits remarkable efficacy through colour transformation of an existing dataset; however, it is crucial to recognise both the advantages and constraints of this technique within clinical applications. This improvement demonstrates potential for enhanced diagnostic precision. The clinical applicability of this method is contingent upon various factors, including the quality and consistency of WLI images, which may differ across diverse devices and imaging conditions. In practical clinical environments, fluctuations in image quality caused by elements like lighting conditions, patient anatomy, and artefacts may influence the efficacy of the SAVE method. Moreover, although the colour transformation markedly improves the spectral information, it may not entirely emulate the functionalities of more sophisticated imaging techniques such as true HSI or NBI, which could provide more comprehensive data for detection. Consequently, the model’s generalisability across diverse clinical settings and patient demographics is a critical factor. Notwithstanding these obstacles, the SAVE method offers a promising strategy for augmenting WLI images without the necessity for specialised equipment, rendering it a cost-effective solution for enhancing non-invasive gastrointestinal diagnostics.

One of the main constraints of this study is the exclusive use of a publicly available dataset and the performance evaluation based only on internal validation [46]. Although the observed results are promising, internal validation alone cannot ensure the generalizability, dependability, and clinical relevance of the suggested SAVE technique. Future studies should focus on doing external validation utilising databases obtained from several hospitals [47]. Such validation will guarantee that the model performs robustly across many imaging situations, patient demographics, and equipment variances, therefore verifying its clinical relevance in practical environments. A multi-institutional dataset including several GIDs is advised to be created. This would enable thorough statistical validation employing techniques including hypothesis testing, confidence interval calculation, and outlier detection, and allow a wider spectrum of clinical traits. While confidence intervals would give a range within which actual population parameters—such as mean accuracy or recall—lie, hypothesis testing would assist in ascertaining whether the observed results are statistically significant. Conversely, outlier detection is crucial for spotting unusual data points, which can be rare clinical situations needing more research or measurement mistakes.

The study’s reliance on quantitative measurements with comparatively less attention to qualitative features, including model interpretability and clinical usability, adds still another significant constraint. The lack of interpretability might make doctors less confident in forecasts produced by artificial intelligence. Future research should thus combine Computer-Aided Diagnosis (CAD) systems with competently offered simple visual explanations like heatmaps, saliency maps, and probability-based overlays [48,49,50]. These interpretability tools would help medical practitioners grasp the thinking of the model, therefore improving diagnosis confidence and supporting clinical decision-making. Furthermore, SAVE faces difficulties with non-linear responses from endoscopic cameras. Often showing a non-linear relationship between light intensity and digital signal output, these cameras produce inaccurate colour representation and varying brightness. Several elements lead to such non-linearity: environmental lighting conditions during image acquisition, onboard image processing techniques, and sensor-specific properties. These distortions limit diagnostic accuracy and reduce the colour quality of the photographs. Dealing with this problem will require the creation of sophisticated calibration methods, especially meant to offset non-linear sensor responses. One future path is the creation of next-generation image sensors with better linearity and spectral accuracy, or alternatively, dedicated non-linear correction algorithms. Moreover, in real-time clinical settings, the computing requirements of SAVE, especially those related to matrix operations like eigenvalue decomposition and PCA, may cause performance constraints. Future research should thus give time complexity of the algorithm top priority, maybe by means of more effective mathematical frameworks and approximation methods. Using parallel computing models like GPU acceleration can greatly improve processing performance, thereby enabling real-time implementation and a more sensible clinical workflow. Another interesting restriction is SAVE’s spectral range, which is presently limited to the visible light spectrum. For sub-surface tissue visualisation, infrared (IR) imaging provides possible advantages encompassing wavelengths between roughly 700 nm and 2500 nm. But compared to visible light sensors, IR sensors usually generate images with reduced spatial resolution and less structural information, which limits their usefulness in uses like GI endoscopy. A small dataset was used to train five pre-trained deep learning models, therefore avoiding too high a computational cost. Turning now to full-scale clinical applications, especially those utilising high-resolution endoscopic video streams, will require significant computational resources. These comprise big memory allotments to enable real-time inference and data storage, as well as high-performance GPUs. Future studies should thus concentrate on creating lightweight, resource-efficient variants of the SAVE technique to handle this. SAVE could be implemented in low-resource or point-of-care settings without sacrificing speed by using model compression methods and effective deep learning architectures.

The SAVE algorithm lowers scalability by means of a human calibration process, therefore adding complexity. Artificial intelligence-driven calibration systems automating this stage would improve usability and guarantee constant image quality. Direct embedding of SAVE into endoscopic devices with automated, real-time calibration features would simplify clinical acceptance and increase diagnosis efficiency. Notwithstanding these difficulties, the SAVE method has great promise to change GID diagnosis. By allowing prompt therapies, lowering the likelihood of complications, and hence saving healthcare expenses, its capacity to precisely characterise GI anomalies at early stages positively influences patient outcomes. Enhanced categorisation accuracy can also lower the number of hospital visits, prevent needless intrusive treatments, and raise patient survival rates generally. SAVE is destined to be a useful tool in enhancing non-invasive, AI-assisted medical diagnostics as the system develops with constant validation and integration. In the future scope of this research, it will be intended to integrate interpretability techniques, such as Grad-CAM or saliency maps, to visualise the areas of interest that deep learning models emphasise during classification. These methods will augment the transparency of AI systems, instilling greater confidence in physicians regarding the model’s decision-making process. By incorporating interpretability techniques, we seek to enhance the reliability and acceptance of artificial intelligence in clinical applications, thereby facilitating its effective utilisation in diagnosing gastrointestinal diseases. The SAVE method will be incorporated into multiple stages of the gastroenterological workflow in clinical practice. During triage, SAVE may be employed to improve the quality of initial endoscopic images, assisting healthcare professionals in prioritising patients according to the severity of their conditions. During the screening phase, SAVE could enhance the identification of early-stage gastrointestinal diseases, such as polyps or ulcers, by delivering superior image quality that uncovers more nuanced abnormalities. Ultimately, during the follow-up phase, SAVE may be utilised to assess disease progression or treatment efficacy by delivering clearer, more detailed images of the gastrointestinal tract, thereby facilitating more informed decision-making. Incorporating SAVE into these phases may augment the diagnostic process, increase efficiency, and ultimately result in improved patient outcomes.

## 5. Conclusions

This study on the validation of SAVE offers a unique method to improve GID detection by the use of NBI and HIS. The SAVE method greatly enhances the visibility and diagnostic accuracy of important GIDs, including ulcers, polyps, and oesophagitis, by using a calibrated transformation process that turns ordinary RGB endoscopic images into spectrally enhanced representations. High-fidelity image transformation with minimum chromatic error is guaranteed by the deployment of a strong colour calibration framework, including the CIE 1931 XYZ colour space and optimal use of regression and PCA. High structural similarity and little perceptual difference between SAVE-generated and genuine NBI images, as shown by experimental results, confirmed the effectiveness of the suggested approach. Furthermore, while this approach shows a scalable end-to-end diagnostic pipeline, the dataset utilised was carefully pre-processed and enriched to facilitate model generalisation. SAVE addresses the restrictions of traditional white light endoscopy and opens the route for more accurate and early-stage detection of GID, therefore reflecting a promising development in non-invasive diagnostic imaging. Future research will investigate clinical environment real-time deployment and extend spectrum calibration for more general medical use.

## Figures and Tables

**Table 1:** Recent advances and the study gap in using VCE.

Study	Dataset Used	Methodology	Originality	Results
[32]	Hyper Kvasir, Kvasir2, CVC-ClinicDB	CNNs (ResNet-50V2, DenseNet-201, VGG-16, RDV-22) combined with SVM and LSTM; K-means for tumour localization	Introduces a multi-phase diagnostic system integrating CNNs with SVM, LSTM, and K-means for colorectal cancer detection	Achieved up to 98.91% accuracy with DenseNet-201; 95.87% accuracy in tumour localisation using K-means
[33]	Kvasir dataset	Spatial-attention ConvMixer architecture for disease classification and detection	Proposes a novel ConvMixer architecture enhanced with spatial attention for improved classification accuracy	Achieved 93.37% accuracy, outperforming other models like ViT and ResNet50
[34]	Kvasir-Capsule dataset	Vision Transformers (ViTs) with TensorFlow Lite quantisation for on-edge deployment	Focuses on deploying ViTs for real-time medical diagnostics on edge devices	Demonstrated effective deployment with reduced model size and maintained performance
[35]	Kvasir-SEG dataset	Deep learning model with encoder-decoder architecture using ConvNeXt and Transformer blocks	Introduces a cross-attention mechanism and Residual Transformer Block for polyp segmentation	Achieved Dice coefficient of 0.8715 and mIoU of 0.8021
Proposed Method	Kvasir dataset	Multiple machine learning models, including EfficientNet, ResNet and VGG 16	Introduced a novel HSI conversion algorithm that can convert any WLI image into an HSI image	Accuracy improvements from 85% (WLI) to 92% (SAVE)

**Table 2:** Dataset specifications.

Class	Train	Validation	Test	Total
Normal	800	500	200	1500
Ulcer	800	500	200	1500
Polyps	800	500	200	1500
Esophagitis	800	500	200	1500
Total	3200	2000	800	6000

**Table 3:** Classification matrix of Efficient Net B2.

Type	Classes	Precision	Recall	F1 Score	Accuracy
WLI	Normal	97%	100%	98%	96%
	Ulcer	96%	92%	94%	
	Polyps	94%	94%	94%	
	Oesophagitis	99%	100%	99%	
SAVE	Normal	98%	100%	99%	97%
	Ulcer	95%	97%	96%	
	Polyps	97%	94%	95%	
	Oesophagitis	100%	100%	100%	

**Table 4:** Classification report of Efficient Net B7.

Type	Classes	Precision	Recall	F1 Score	Accuracy
WLI	Normal	98%	100%	99%	97%
	Ulcer	99%	92%	95%	
	Polyps	95%	97%	96%	
	Oesophagitis	97%	100%	98%	
SAVE	Normal	100%	100%	100%	98%
	Ulcer	97%	94%	95%	
	Polyps	94%	97%	96%	
	Oesophagitis	99%	100%	100%	

**Table 5:** Classification report of ResNet 50.

Type	Classes	Precision	Recall	F1 Score	Accuracy
WLI%	Normal	98%	100%	99%	92%
	Ulcer	98%	72%	83%	
	Polyps	80%	97%	87%	
	Oesophagitis	98%	100%	99%	
SAVE	Normal	97%	100%	98%	95%
	Ulcer	89%	96%	93%	
	Polyps	97%	85%	91%	
	Oesophagitis	99%	100%	99%	

**Table 6:** Classification report of ResNet 101.

Type	Classes	Precision	Recall	F1-Score	Accuracy
WLI	Normal	98%	100%	99%	93%
	Ulcer	96%	76%	85%	
	Polyps	82%	95%	88%	
	Oesophagitis	98%	100%	99%	
SAVE	Normal	100%	100%	100%	97%
	Ulcer	94%	95%	95%	
	Polyps	96%	94%	95%	
	Oesophagitis	100%	100%	100%	

**Table 7:** Classification result of VGG16.

Type	Classes	Precision	Recall	F1-Score	Accuracy
WLI	Normal	86%	100%	92%	85%
	Ulcer	96%	54%	69%	
	Polyps	71%	89%	79%	
	Oesophagitis	98%	99%	99%	
SAVE	Normal	94%	100%	97%	95%
	Ulcer	96%	88%	91%	
	Polyps	91%	93%	92%	
	Oesophagitis	99%	100%	100%	

## Data Availability

The data presented in this study are available in this article upon considerable request to the corresponding author (H.-C.W.).

## Supplementary Materials

The following supporting information can be downloaded at: https://www.mdpi.com/article/10.3390/diagnostics15131664/s1, Figure S1. Confusion matrix and classification report of WLI Efficient Net B2; Figure S2. Confusion matrix and classification report of SAVE Efficient Net B2; Figure S3. Confusion matrix and classification report of WLI Efficient Net B7; Figure S4. Confusion matrix and classification report of SAVE Efficient Net B7; Figure S5. Confusion matrix and classification report of WLI Resnet 50; Figure S6. Confusion matrix and classification report of SAVE Resnet 50; Figure S7. Confusion matrix and classification report of WLI Resnet 101; Figure S8. Confusion matrix and classification report of SAVE Resnet 101; Figure S9. Confusion matrix and classification report of WLI VGG16; Figure S10. Confusion matrix and classification report of SAVE VGG16; Table S1. Hyperparameter of the models used; Table S2. RMSEs of the XYZ values before and after calibration; Figure S11. The color difference before and after camera calibration; Figure S12. RMSEs between analog and measured spectra of each color block; Figure S13. LAB values of the simulated and observed colors.
