# Supplementary material for: Software-Based Transformation of White Light Endoscopy Images to Hyperspectral Images for Improved Gastrointestinal Disease Detection
Source: Diagnostics (Basel). 2025 Jun 30;15(13):1664. doi: 10.3390/diagnostics15131664 (PMC12248717; doi:10.3390/diagnostics15131664)
Supplement: Supplementary file 1 [file diagnostics-15-01664-s001.zip › diagnostics-3660147-supplementary.pdf]

*diagnostics*

MDPI

*Article*

# Software-Based Transformation of White Light Endoscopy Images to Hyperspectral Images for Improved Gastrointestinal Disease Detection: Supplementary

**Chien-Wei Huang [1,2], Chang-Chao Su [3], Chu-Kuang Chou [3,4], Arvind Mukundan [5], Riya Karmakar [5], Tsung-Hsien Chen [6], Pranav Shukla [7], Devansh Gupta [8] and Hsiang-Chen Wang [5,9,10,*]**

1 Department of Gastroenterology, Kaohsiung Armed Forces General Hospital, 2, Zhongzheng 1st Rd., Lingya District, Kaohsiung 80284, Taiwan; forevershiningfy@yahoo.com.tw
2 Department of Nursing, Tajen University, 20, Weixin Rd., Yanpu Township, Pingtung County 90741, Taiwan
3 Division of Gastroenterology and Hepatology, Department of Internal Medicine, Ditmanson Medical Foundation Chia-Yi Christian Hospital, Chiayi 60002, Taiwan; 06155@cych.org.tw (C.-C.S.); vacinu@gmail.com (C.-K.C.)
4 Obesity Center, Ditmanson Medical Foundation Chia-Yi Christian Hospital, Chiayi 60002, Taiwan
5 Department of Mechanical Engineering, National Chung Cheng University, 168, University Rd., Min Hsiung, Chiayi 62102, Taiwan; d09420003@ccu.edu.tw (A.M.); karmakarriya345@gmail.com (R.K.)
6 Department of Internal Medicine, Ditmanson Medical Foundation Chia-Yi Christian Hospital, Chiayi 60002, Taiwan; cych13794@gmail.com
7 Department of Computer Science, Sanjivani College of Engineering, Station Rd., Singapur, Kopargaon 423603, Maharashtra, India; pranavshukla5467@gmail.com
8 Computer Science and Engineering Department, Thapar Institute of Engineering & Technology, Patiala 147001, Punjab, India; dgupta1be21@thapar.edu
9 Department of Medical Research, Dalin Tzu Chi Hospital, Buddhist Tzu Chi Medical Foundation, No. 2, Minsheng Road, Dalin, Chiayi 62247, Taiwan
10 Hitspectra Intelligent Technology Co., Ltd., Kaohsiung 80661, Taiwan
* Correspondence: hcwang@ccu.edu.tw

Academic Editor: Gemma Piella

Received: 8 May 2025
Revised: 24 June 2025
Accepted: 25 June 2025
Published: 30 June 2025

**Citation:** Huang, C.-W.; Su, C.-C.; Chou, C.-K.; Mukundan, A.; Karmakar, R.; Chen, T.-H.; Shukla, P.; Gupta, D.; Wang, H.-C. Software-Based Transformation of White Light Endoscopy Images to Hyperspectral Images for Improved Gastrointestinal Disease Detection. *Diagnostics* **2025**, *15*, 1664. https://doi.org/10.3390/diagnostics15131664

**Copyright:** © 2025 by the authors. Submitted for possible open-access publication under the terms and conditions of the Creative Commons Attribution (CC BY) license (https://creativecommons.org/licenses/by/4.0/).

## S1. Dataset

Two publicly available datasets used for the classification of GI disease. First one is KVASIR dataset which is the first multi-class dataset for detection and classification of GI tract disease and the dataset was collected and verified by experienced gastroenterologists from Vestre Viken Health Trust, consists of image captured during the Wireless Capsule Endoscopy (WCE) procedure which is widely practiced in real-world endoscopic examinations . Second one is ETIS-Larib Polyp DB dataset. In this work, the curation of these datasets occurred in a stochastic fashion with proper awareness to prevent data leakage and added bias. The curated dataset with image samples labeled with their proper classes, quantity, and distribution. The prepared dataset has 6,000 images which is further divided into 3-part train, validation, and test, which contained 3,200, 2,000, and 800 shown in Tabel 1. The dimensions of images get vary among the dataset. Therefore, an automated image generator from Keras was used to resized the train images to a fixed dimension of 224 × 224. This method had improved the allocation of resources, increased training speeds, and prevented the depletion of computing memory during experiments. Nonetheless, such can expand depending on the given machine specification.

## S2. Deep learning Models

### *S2.1 EfficientNetB2*

Figure S1. Confusion matrix and classification report of WLI Efficient Net B2

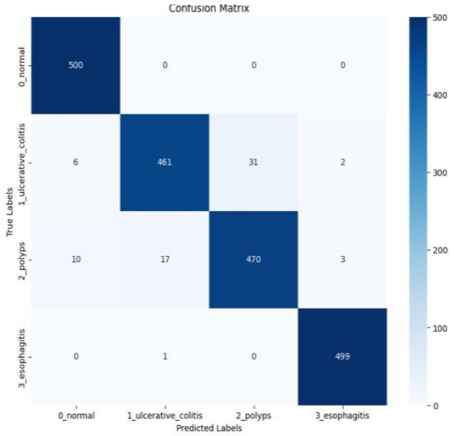


| | precision | recall | f1-score | support |
|---|---|---|---|---|
| 0_normal | 0.97 | 1.00 | 0.98 | 500 |
| 1_ulcerative_colitis | 0.96 | 0.92 | 0.94 | 500 |
| 2_polyps | 0.94 | 0.94 | 0.94 | 500 |
| 3_esophagitis | 0.99 | 1.00 | 0.99 | 500 |
| accuracy | | | 0.96 | 2000 |
| macro avg | 0.96 | 0.97 | 0.96 | 2000 |
| weighted avg | 0.96 | 0.96 | 0.96 | 2000 |

Figure S2. Confusion matrix and classification report of SAVE Efficient Net B2

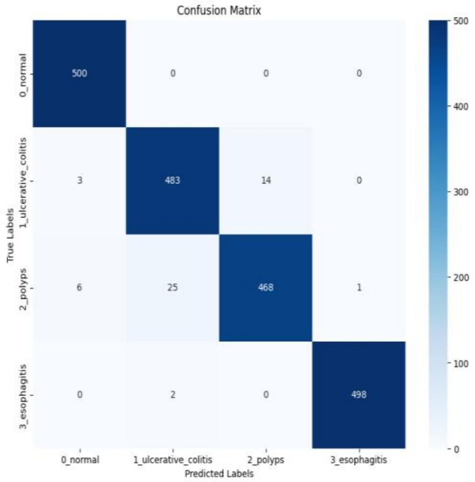


| | precision | recall | f1-score | support |
|---|---|---|---|---|
| 0_normal | 0.98 | 1.00 | 0.99 | 500 |
| 1_ulcerative_colitis | 0.95 | 0.97 | 0.96 | 500 |
| 2_polyps | 0.97 | 0.94 | 0.95 | 500 |
| 3_esophagitis | 1.00 | 1.00 | 1.00 | 500 |
| accuracy | | | 0.97 | 2000 |
| macro avg | 0.97 | 0.97 | 0.97 | 2000 |
| weighted avg | 0.97 | 0.97 | 0.97 | 2000 |

### *S2.2 EfficientNetB7*

Figure S3. Confusion matrix and classification report of WLI Efficient Net B7

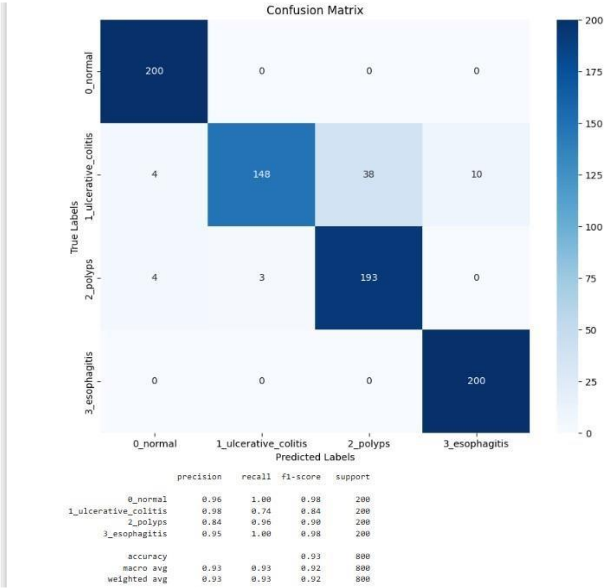


Figure S4. Confusion matrix and classification report of SAVE Efficient Net B7

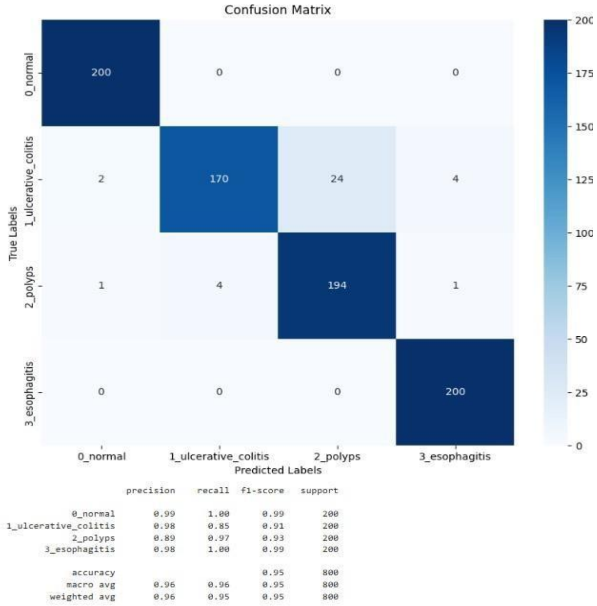


*S2.3 Resnet 50*

Figure S5. Confusion matrix and classification report of WLI Resnet 50

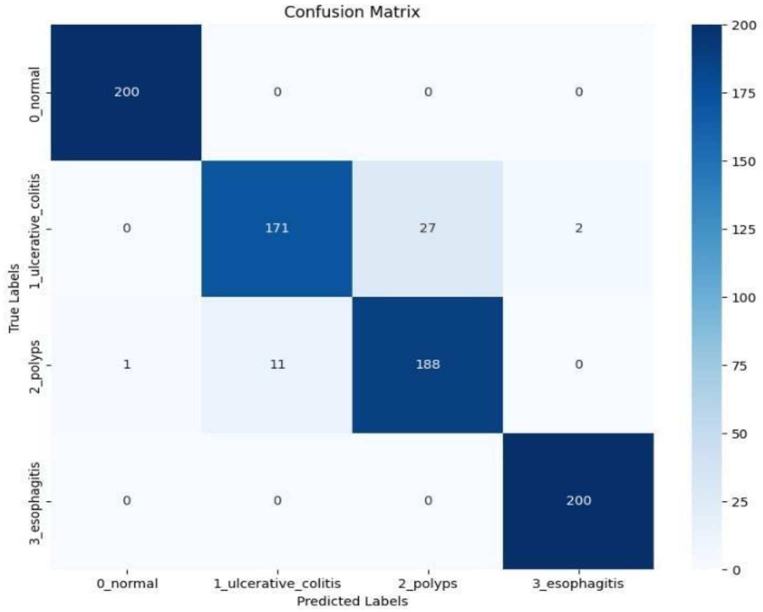


| | precision | recall | f1-score | support |
|---|---|---|---|---|
| 0_normal | 1.00 | 1.00 | 1.00 | 200 |
| 1_ulcerative_colitis | 0.94 | 0.85 | 0.90 | 200 |
| 2_polyps | 0.87 | 0.94 | 0.91 | 200 |
| 3_esophagitis | 0.99 | 1.00 | 1.00 | 200 |
| accuracy | | | 0.95 | 800 |
| macro avg | 0.95 | 0.95 | 0.95 | 800 |
| weighted avg | 0.95 | 0.95 | 0.95 | 800 |

Figure S6. Confusion matrix and classification report of SAVE Resnet 50

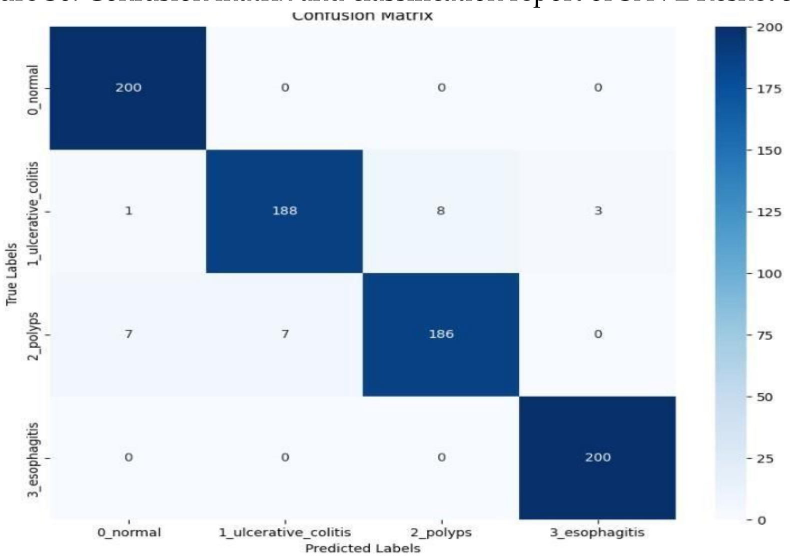


| | precision | recall | f1-score | support |
|---|---|---|---|---|
| 0_normal | 0.96 | 1.00 | 0.98 | 200 |
| 1_ulcerative_colitis | 0.96 | 0.94 | 0.95 | 200 |
| 2_polyps | 0.96 | 0.93 | 0.94 | 200 |
| 3_esophagitis | 0.99 | 1.00 | 0.99 | 200 |
| accuracy | | | 0.97 | 800 |
| macro avg | 0.97 | 0.97 | 0.97 | 800 |
| weighted avg | 0.97 | 0.97 | 0.97 | 800 |

*S2.4 Resnet 101*

Figure S7. Confusion matrix and classification report of WLI Resnet 101

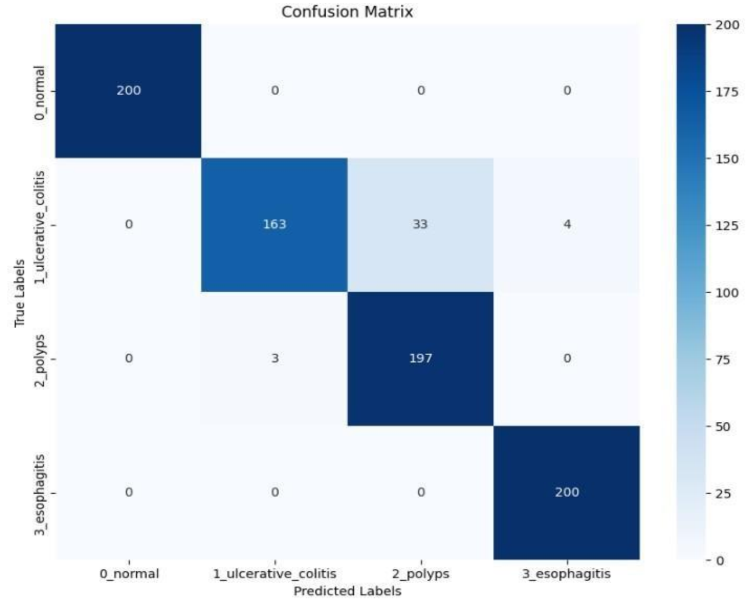


| | precision | recall | f1-score | support |
|---|---|---|---|---|
| 0_normal | 1.00 | 1.00 | 1.00 | 200 |
| 1_ulcerative_colitis | 0.98 | 0.81 | 0.89 | 200 |
| 2_polyps | 0.86 | 0.98 | 0.92 | 200 |
| 3_esophagitis | 0.98 | 1.00 | 0.99 | 200 |
| accuracy | | | 0.95 | 800 |
| macro avg | 0.95 | 0.95 | 0.95 | 800 |
| weighted avg | 0.95 | 0.95 | 0.95 | 800 |

Figure S8. Confusion matrix and classification report of SAVE Resnet 101

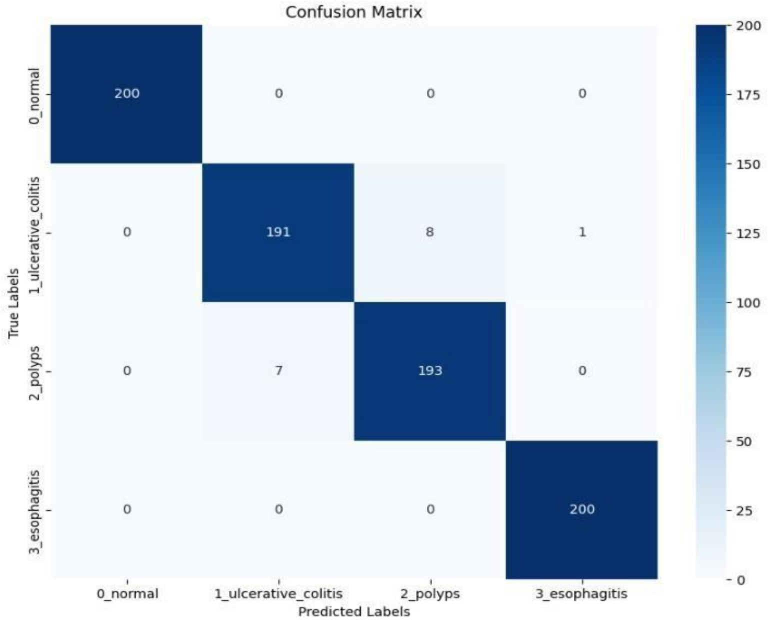


| | precision | recall | f1-score | support |
|---|---|---|---|---|
| 0_normal | 1.00 | 1.00 | 1.00 | 200 |
| 1_ulcerative_colitis | 0.96 | 0.95 | 0.96 | 200 |
| 2_polyps | 0.96 | 0.96 | 0.96 | 200 |
| 3_esophagitis | 1.00 | 1.00 | 1.00 | 200 |
| accuracy | | | 0.98 | 800 |
| macro avg | 0.98 | 0.98 | 0.98 | 800 |
| weighted avg | 0.98 | 0.98 | 0.98 | 800 |

*S2.5 VGG 16*

Figure S9. Confusion matrix and classification report of WLI VGG16

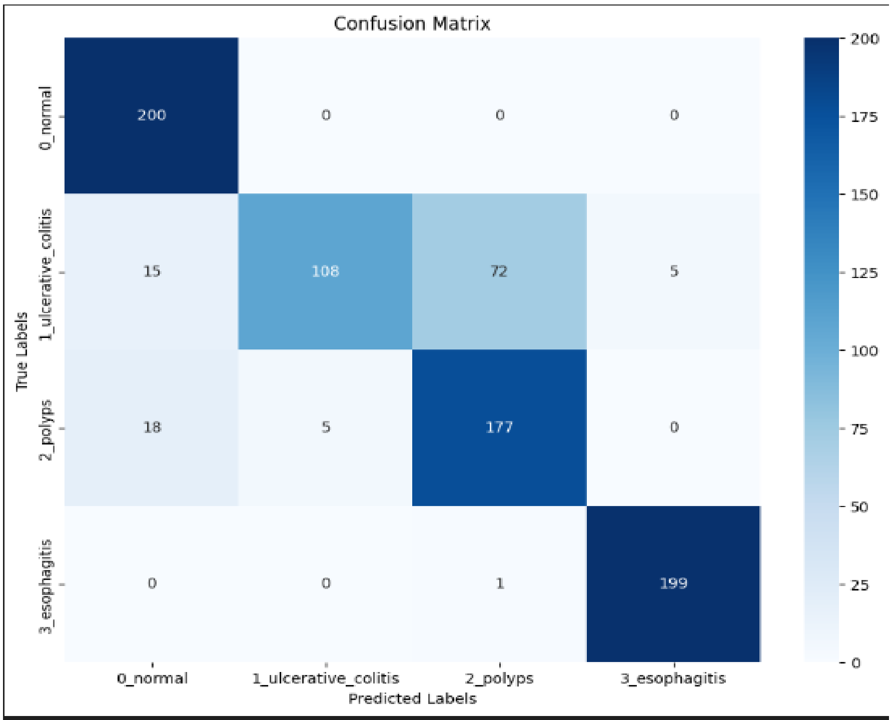


| | precision | recall | f1-score | support |
|---|---|---|---|---|
| 0_normal | 0.86 | 1.00 | 0.92 | 200 |
| 1_ulcerative_colitis | 0.96 | 0.54 | 0.69 | 200 |
| 2_polyps | 0.71 | 0.89 | 0.79 | 200 |
| 3_esophagitis | 0.98 | 0.99 | 0.99 | 200 |
| accuracy | | | 0.85 | 800 |
| macro avg | 0.87 | 0.85 | 0.85 | 800 |
| weighted avg | 0.87 | 0.85 | 0.85 | 800 |

Figure S10. Confusion matrix and classification report of SAVE VGG16

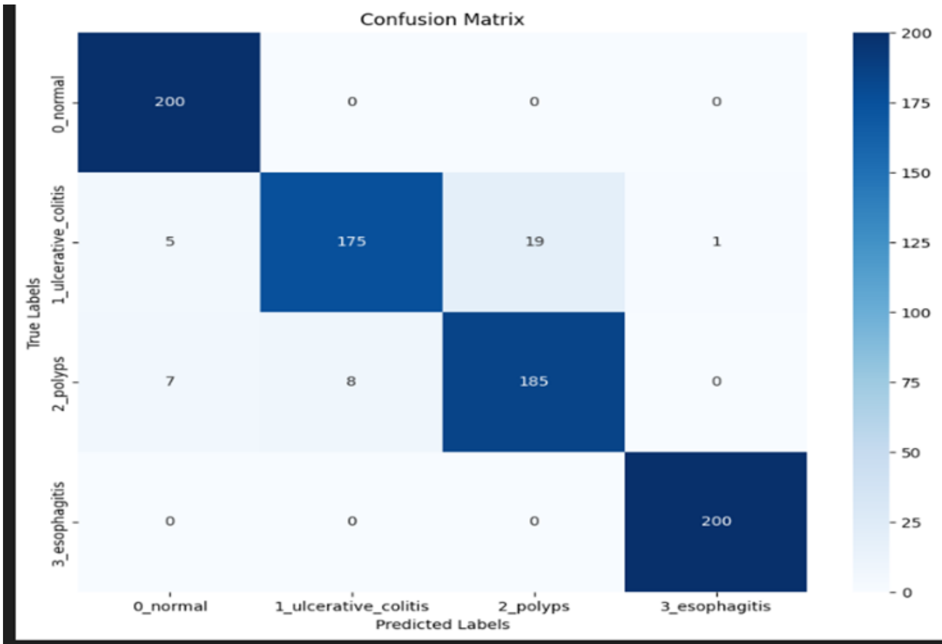


| | precision | recall | f1-score | support |
|---|---|---|---|---|
| 0_normal | 0.94 | 1.00 | 0.97 | 200 |
| 1_ulcerative_colitis | 0.96 | 0.88 | 0.91 | 200 |
| 2_polyps | 0.91 | 0.93 | 0.92 | 200 |
| 3_esophagitis | 1.00 | 1.00 | 1.00 | 200 |
| accuracy | | | 0.95 | 800 |
| macro avg | 0.95 | 0.95 | 0.95 | 800 |
| weighted avg | 0.95 | 0.95 | 0.95 | 800 |

**Hardware and Software Specifications:**

- Hardware: The classification models were trained and evaluated on a system equipped with the following hardware:
- Processor: [Intel Core i5 ™ 10500K]
- GPU: [NVIDIA GeForce RTX 3060]
- RAM: [RAM 16 GB]
- Storage: [ SSD with 1 TB capacity]
- Software:
- Operating System: [Windows 11, 10.0.26100]

Table S1. Hyperparameter of the models used.

| Model | Hyperparameter | Value/Range |
|---|---|---|
| EfficientNet | Learning Rate | 0.001 |
| | Batch Size | 32 |
| | Epochs | 300 |
| | Optimizer | Adam |
| ResNet50 | Learning Rate | 0.001 |
| | Batch Size | 32 |
| | Epochs | 300 |
| | Optimizer | SGD with momentum |
| VGG16 | Learning Rate | 0.001 |
| | Batch Size | 32 |
| | Epochs | 300 |
| | Optimizer | Adam |

## S3. Evaluation Metrices

The evaluation metrics included in this particular study are Precision (P), Recall (R), mean Average Precision (mAP), F1-score and confusion matrix. Some of the concepts associated with these metrics are True Positive (TP), False Positive (FP), False Negative (FN). TP, just as the name suggests, are correct detection of ground truth bounding box. While FP are incorrect detections of a nonexciting class or misplaced detection of an object and FN are simply undetected ground-truth bounding box.

Precision is an evaluation metric that measures the number of instances that were correctly predicted. It is given by equation (S1):

$$P = \left(\frac{TP}{TP+FP}\right) \times 100 \quad \text{(S1)}$$

High precision value indicates that the model has low rate of false positives. Having high precision reduces the chances of misclassifying any skin

Recall is known as positive rate and is a probability of actual positive instances that model identifies correctly. It is given by equation (S2):

$$R = \left(\frac{TP}{TP+FN}\right) \times 100 \quad \text{(S2)}$$

F1-score is measure of predictive performance. It is calculated based on precision and recall of the model and is given by:

$$F1 = \frac{2 \times Precision \times Recall}{Precision + Recall} \quad \text{(S3)}$$

The area under the precision-recall curve for a specific class in precision vs recall graph plotted for different threshold values is the average precision (AP). mAP is the mean of APs and is given by equation (S4):

$$AP = \int_0^1 Precision(Recall)\, dRecall \quad \text{(S4)}$$

## S4. SAVE Results

The data generated through simulation were utilized for the initial evaluation of the system's performance. The characterization involved measuring their spectrum emissions, while also taking into account the sensitivities provided by the camera manufacturers. The spectral curves of the 24-color Macbeth Color Checker chart were utilized for both the training and validation sets of the samples. Table S1 displays the RMSE measurements for each of the 24 hues.

Table S2. RMSEs of the XYZ values before and after calibration.

| S.no | Before calibration | | | After Calibration | | | RMSE | SD |
|---|---|---|---|---|---|---|---|---|
| | X | Y | Z | X | Y | Z | | |
| 1 | 10.96 | 9.92 | 4.63 | 11.14 | 9.87 | 4.26 | 0.24 | 0.30 |
| 2 | 38.74 | 35.80 | 18.65 | 38.57 | 35.94 | 18.66 | 0.13 | 0.08 |
| 3 | 16.62 | 19.07 | 24.13 | 16.48 | 18.79 | 24.11 | 0.18 | 0.17 |
| 4 | 10.33 | 12.86 | 4.62 | 10.16 | 13.03 | 4.85 | 0.19 | 0.19 |
| 5 | 24.05 | 23.87 | 31.55 | 24.16 | 24.07 | 31.60 | 0.13 | 0.08 |
| 6 | 30.12 | 42.15 | 32.40 | 30.10 | 42.17 | 32.42 | 0.02 | 0.002 |
| 7 | 38.10 | 30.24 | 4.28 | 38.04 | 30.37 | 4.22 | 0.09 | 0.04 |
| 8 | 11.70 | 11.47 | 25.90 | 11.64 | 11.37 | 25.91 | 0.07 | 0.02 |
| 9 | 29.01 | 19.91 | 9.62 | 29.20 | 19.78 | 9.60 | 0.13 | 0.08 |
| 10 | 8.26 | 6.49 | 9.63 | 8.06 | 6.49 | 9.86 | 0.18 | 0.17 |
| 11 | 34.15 | 44.06 | 8.44 | 34.15 | 44.02 | 8.53 | 0.06 | 0.01 |
| 12 | 47.99 | 44.55 | 6.05 | 48.05 | 44.34 | 6.17 | 0.15 | 0.11 |
| 13 | 6.82 | 5.79 | 21.07 | 6.90 | 5.91 | 21.00 | 0.09 | 0.04 |
| 14 | 14.55 | 23.55 | 7.22 | 14.58 | 23.51 | 7.12 | 0.07 | 0.02 |
| 15 | 21.08 | 12.25 | 3.57 | 21.01 | 12.28 | 3.65 | 0.06 | 0.01 |
| 16 | 58.40 | 60.69 | 7.54 | 58.38 | 60.79 | 7.42 | 0.09 | 0.04 |
| 17 | 28.98 | 19.54 | 20.67 | 28.94 | 19.52 | 20.66 | 0.02 | 0.002 |
| 18 | 12.81 | 19.01 | 28.54 | 12.84 | 19.10 | 28.56 | 0.05 | 0.01 |
| 19 | 82.12 | 88.54 | 67.20 | 82.31 | 88.73 | 67.51 | 0.24 | 0.30 |
| 20 | 54.74 | 58.92 | 45.52 | 54.28 | 58.40 | 44.75 | 0.60 | 1.89 |
| 21 | 33.08 | 35.73 | 27.24 | 33.26 | 35.82 | 27.54 | 0.21 | 0.23 |
| 22 | 18.18 | 19.62 | 14.94 | 18.86 | 20.31 | 15.62 | 0.68 | 2.43 |
| 23 | 9.13 | 10.01 | 8.13 | 8.56 | 9.26 | 7.21 | 0.76 | 3.04 |
| 24 | 2.87 | 3.19 | 2.39 | 3.10 | 3.35 | 2.68 | 0.23 | 0.27 |
| | | | Average | | | | 0.19 | 0.39 |

The calibration of the camera is a crucial component of the SAVE algorithm. Figure S12 displays the color disparity outcomes prior to and following calibration. Following the calibration of the camera, the color exhibited a striking resemblance to the color acquired by the spectrum analyzer, rendering the distinction challenging to perceive. Prior to camera calibration, the mean chromatic aberration of all 24 color blocks was 10.76. After calibrating the camera, the average chromatic aberration decreased to a minimum of 0.63.

| S.no | Before Camera Calibration | Spectrometer | Chromatic Aberration | After Camera Calibration | Spectrometer | Chromatic Aberration |
|---|---|---|---|---|---|---|
| 1 | | | 7.08 | | | 1.24 |
| 2 | | | 7.63 | | | 0.78 |
| 3 | | | 16.43 | | | 0.86 |
| 4 | | | 12.45 | | | 1.68 |
| 5 | | | 14.92 | | | 0.45 |
| 6 | | | 10.80 | | | 0.05 |
| 7 | | | 7.47 | | | 0.52 |
| 8 | | | 18.46 | | | 0.22 |
| 9 | | | 13.19 | | | 0.62 |
| 10 | | | 8.09 | | | 1.30 |
| 11 | | | 8.03 | | | 0.09 |
| 12 | | | 6.43 | | | 0.58 |
| 13 | | | 10.32 | | | 0.30 |
| 14 | | | 12.19 | | | 0.23 |
| 15 | | | 13.31 | | | 0.17 |
| 16 | | | 7.00 | | | 0.18 |
| 17 | | | 17.80 | | | 0.03 |
| 18 | | | 22.22 | | | 0.19 |
| 19 | | | 0.00 | | | 0.08 |
| 20 | | | 5.30 | | | 0.30 |
| 21 | | | 9.77 | | | 0.42 |
| 22 | | | 12.71 | | | 0.81 |
| 23 | | | 13.34 | | | 2.01 |
| 24 | | | 3.37 | | | 1.96 |
| | Average Color Difference | | 10.76 | Average Color Difference | | 0.63 |

Figure S11. The color difference before and after camera calibration

In Figure S12, the reflectance values of the six primary colors inside the 24-color block are illustrated. These colors are blue (13), red (15), green (14), yellow (16), magenta (17), and cyan (18). Based on the analysis of the 24-color blocks, it was noted that the red block

exhibited the most significant disparity between the simulated and actual reflectance values, particularly across the longer wavelength range of 600 to 780 nm. One of the limitations of the study is considered to be this element. All of the remaining 23 color blocks exhibited RMSEs below 0.1, with the color black demonstrating the lowest RMSE of 0.015. The RMSE was merely 0.056, suggesting that the majority of the color could be replicated with precision.

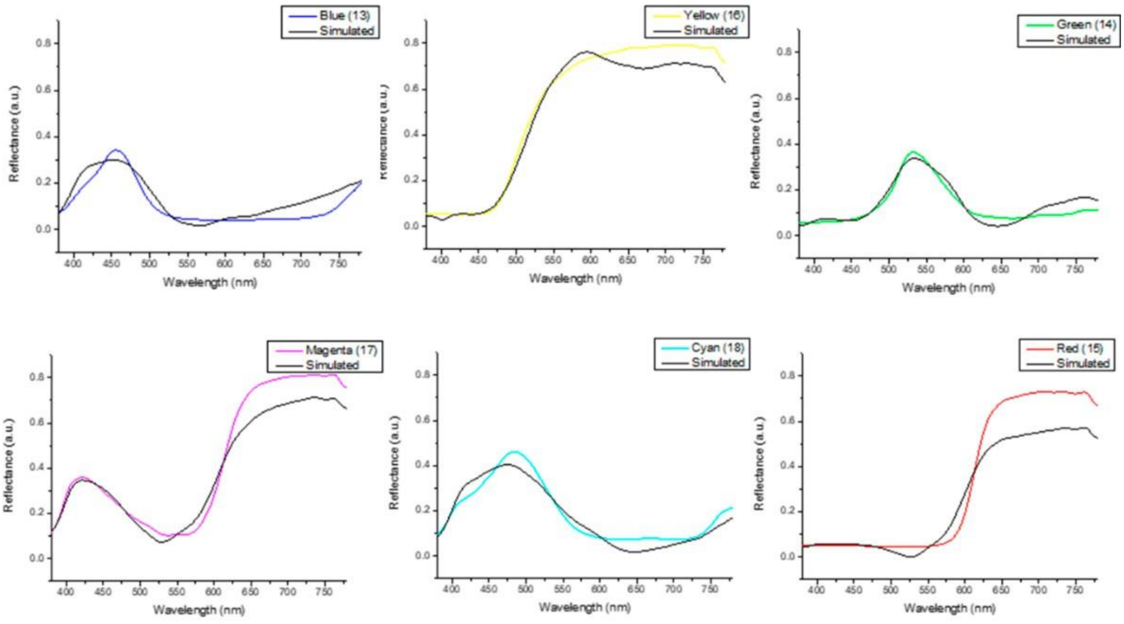


Figure S12. RMSEs between analog and measured spectra of each color block

RMSE values can be graphically and mathematically represented by calculating the disparity between simulated and measured colors. The representation of a color can be denoted as LAB, where L represents lightness, channel A, and channel B, respectively. The numerical definition of any color can be achieved by manipulating the values of L, A, and B. The L, A, and B values of the simulated and computed colors are depicted in Figure S14. The average color disparity was about 0.75, suggesting that the replicated color was visually precise.

| Measured Color | | | | Simulated Color | | | | Color Difference |
|---|---|---|---|---|---|---|---|---|
| L | a | b | Color | L | a | b | Color | |
| 37.61 | 13.65 | 24.56 | | 37.64 | 11.87 | 22.60 | | 1.32 |
| 66.48 | 14.68 | 31.10 | | 66.37 | 15.47 | 30.58 | | 0.73 |
| 50.44 | -7.58 | -6.44 | | 50.72 | -8.16 | -6.09 | | 0.75 |
| 42.80 | -16.14 | 30.50 | | 42.48 | -14.04 | 31.19 | | 1.50 |
| 56.16 | 5.70 | -8.01 | | 55.96 | 5.89 | -8.51 | | 0.43 |
| 70.99 | -34.14 | 16.44 | | 71.01 | -34.00 | 15.65 | | 0.41 |
| 61.97 | 32.36 | 66.76 | | 61.85 | 33.07 | 65.32 | | 0.80 |
| 40.20 | 6.07 | -27.03 | | 40.27 | 6.15 | -26.67 | | 0.27 |
| 51.59 | 46.04 | 27.52 | | 51.72 | 44.60 | 27.21 | | 0.52 |
| 30.62 | 18.70 | -9.45 | | 30.52 | 20.85 | -8.70 | | 1.46 |
| 72.24 | -24.91 | 66.55 | | 72.23 | -25.21 | 66.79 | | 0.13 |
| 72.46 | 17.04 | 75.67 | | 72.59 | 16.33 | 76.17 | | 0.49 |
| 29.18 | 13.90 | -37.66 | | 28.70 | 15.23 | -38.14 | | 0.81 |
| 55.59 | -40.93 | 42.88 | | 55.56 | -41.82 | 42.54 | | 0.42 |
| 41.66 | 53.78 | 34.95 | | 41.60 | 54.34 | 34.25 | | 0.51 |
| 82.26 | 1.48 | 87.73 | | 82.21 | 1.96 | 87.67 | | 0.27 |
| 51.29 | 46.36 | 1.08 | | 51.30 | 46.12 | 0.95 | | 0.10 |
| 50.80 | -31.41 | -12.85 | | 50.66 | -30.83 | -13.20 | | 0.42 |
| 95.47 | -3.88 | 21.64 | | 95.38 | -3.70 | 22.54 | | 0.52 |
| 80.96 | -3.08 | 18.47 | | 81.32 | -3.39 | 17.74 | | 0.63 |
| 66.38 | -2.74 | 15.56 | | 66.33 | -3.33 | 15.44 | | 0.69 |
| 52.18 | -2.26 | 12.86 | | 51.36 | -2.59 | 12.85 | | 0.91 |
| 36.47 | -2.05 | 9.55 | | 37.78 | -3.18 | 8.64 | | 2.00 |
| 21.40 | -1.45 | 6.28 | | 20.70 | -2.87 | 7.50 | | 2.02 |
| | | | | | | | Average Color Difference | 0. 75 |

Figure S13. LAB values of the simulated and observed colors.

**Author Contributions:** Conceptualization, D.G., C.-K.C., and H.-C.W.; methodology, D.G., C.-K.C., and R.K.; software, D.G.; validation, D.G., C.-K.C., H.-C.W., and C.-C.S.; formal analysis, D.G.; investigation, D.G., R.K., and A.M.; resources, C.-K.C. and C.-W.H.; data curation, D.G.; writing—original draft preparation, D.G., and T.-H.C,; writing—review and editing, D.G., T.-H.C., C.-K.C., and H.-C.W.; visualization, D.G.; supervision, H.-C.W., T.-H.C, and C.-W.H.; project administration, H.-C.W.; funding acquisition, C.-W.H. and H.-C.W. All authors have read and agreed to the published version of the manuscript.

**Funding:** This research was supported by the National Science and Technology Council, the Republic of China, under grants NSTC 113-2221-E-194-011-MY3. This study was financially or partially supported by the Dalin Tzu Chi Hospital, Buddhist Tzu Chi Medical Foundation-National Chung Cheng University Joint Research Program and Kaohsiung Armed Forces General Hospital Research Program KAFGH_D_114012 in Taiwan.

**Institutional Review Board Statement:** The study was conducted according to the guidelines of the Declaration of Helsinki and approved by the Institutional Review Board of the Institutional Review Board of Kaohsiung Armed Forces General Hospital (KAFGHIRB 114-022).

**Informed Consent Statement:** Written informed consent was waived in this study because of the retrospective, anonymized nature of study design.

**Data Availability Statement:** The data presented in this study are available in this article upon considerable request to the corresponding author (H.-C.W.).

**Conflicts of Interest:** Author Hsiang-ChenWang was employed by the company Hit spectra Intelligent Technology Co., Ltd. The remaining authors declare that the research was conducted in the absence of any commercial or financial relationships that could be construed as a potential conflict of interest.

**Disclaimer/Publisher's Note:** The statements, opinions and data contained in all publications are solely those of the individual author(s) and contributor(s) and not of MDPI and/or the editor(s). MDPI and/or the editor(s) disclaim responsibility for any injury to people or property resulting from any ideas, methods, instructions or products referred to in the content.
